# Invasion status stratifies the composition of the human pancreatic cancer perineural niche

**DOI:** 10.64898/2026.08.05.743048

**Published:** 2026-08-06

**Authors:** Zainab Hussain, Dig Vijay Kumar Yarlagadda, Qianzi Li, Nuray Tezcan, Pavel Stupakov, Golabahar Sadatrezaei, Richard J. Wong, Joan Massagué, Zeynep Tarcan, Olca Basturk, Sylvie Deborde, Christina S. Leslie, Mara H. Sherman

## Abstract

The peripheral nervous system innervates the pancreatic ductal adenocarcinoma (PDAC) microenvironment, and perineural invasion (PNI), the invasion of cancer cells in and around nerves, correlates with metastatic burden and poor outcomes. Though PDAC innervation is near universal and the majority of PDAC patients harbor PNI, the cellular and molecular composition of the heterocellular perineural niche is largely unknown, obscuring functional significance. Here we provide a deeply phenotyped spatial and single-cell atlas of the human PDAC perineural niche, enabling a high-resolution comparison of invaded versus non-invaded nerve neighborhoods. This atlas leverages a novel vision-centric artificial intelligence model for imaging-based spatial transcriptomics, coupled with pathology-guided single-nucleus RNA-seq. These analyses revealed that invaded nerve neighborhoods harbor cancer cells of the classical subtype, together with myofibroblastic cancer-associated fibroblasts (CAFs) and lipid-associated macrophages. Non-invaded nerve neighborhoods, in contrast, harbor inflammatory CAFs and infiltration of B and T lymphocytes. These findings raise the possibility that PNI and not innervation itself is immune-suppressive in this setting, motivating functional studies.

**Statement of Significance:**

Human PDAC is innervated and almost invariably harbors PNI, but the composition and functions of perineural niches remain unclear. Here we provide a spatial and single-cell atlas of the human PDAC perineural niche, defining cancer and stromal cell states in the context of PNI and nominating this process as potentially immune-suppressive.

## Full Text Availability

The license terms selected by the author(s) for this preprint version do not permit archiving in PMC. The full text is available from the preprint server.

